# The Relationship Between Fetal Central Nervous System Malformations and Modified Myocardial Performance Index

**DOI:** 10.7759/cureus.47287

**Published:** 2023-10-18

**Authors:** Gokce Naz Kucukbas, Mustafa Bağcı, Hanım Güler Şahin

**Affiliations:** 1 Obstetrics and Gynecology/Perinatology, Kocaeli City Hospital, Kocaeli, TUR; 2 Obstetrics and Gynecology, Van Yüzüncü Yıl University, Van, TUR

**Keywords:** calvarium defects, cardiac function, fetus, modified myocardial performance index, central nervous system malformation

## Abstract

Introduction: Fetal ventriculomegaly, the most commonly identified abnormality of the fetal central nervous system (CNS), has been associated with elevated levels of the modified myocardial performance index (mMPI). However, the impact of other CNS pathologies on mMPI has not yet been evaluated. This study aimed to investigate whether there were changes in the myocardial performance index of fetuses with CNS pathologies without congenital heart diseases.

Methods: A total of 126 singleton pregnant women were included in this study. Sixty-three fetuses had fetal CNS abnormalities of acrania, anencephaly, encephalocele, Dandy-Walker malformation, hydrocephalus, and meningocele. The control group consisted of 63 healthy and gestational age-matched fetuses. All ultrasonographic examinations were done in the second trimester of gestation. The data related to the characteristics of pregnant women were evaluated, and fetal left ventricular mMPI was obtained by ultrasound scan.

Results: The study and the control group participants were not significantly different by means of pregnancy characteristics. The mean mMPI was higher in the fetal CNS malformation group compared to the control groups (0.39±0.02 vs. 0.45±0.04, P<0.001). The mean mMPI value was similar for fetuses with both closed and open calvarium defects of fetal CNS malformation.

Conclusion: Fetal CNS anomalies may be associated with prenatal cardiac dysfunction. Moreover, this relationship might be independent of the type of fetal CNS malformation, whether a closed or open calvarium defect.

## Introduction

Ultrasonographic evaluation of cardiac function is gaining prominence as it allows for the nuanced identification of myocardial dysfunction during development, potentially guiding intervention strategies to enhance neonatal outcomes [1]. In fetal cardiology, the myocardial performance index (MPI), derived by dividing the sum of the isovolumetric contraction time (IVCT) and the isovolumetric relaxation time (IVRT) by the ejection time (ET), is an ultrasound-based metric used for assessing global myocardial function [2,3]. Besides, the modified MPI (mMPI) based on the closing and opening clicks of the aortic and mitral valves in Doppler ultrasonography improved both intraobserver and interobserver reliability and repeatability [4,5].

Serial measurement of MPI during pregnancy has been shown to reflect the maturation or developmental changes of myocardial performance in utero [6]. Given the pivotal role of the central nervous system (CNS) in circulatory regulation, aberrations in the fetal central nervous system can culminate in altered cardiac functionality. The CNS orchestrates cardiovascular functions through the autonomic nervous system, even in utero [7]. Among these regulatory mechanisms, there is a notable adaptation under extended ischemic conditions seen in fetal growth restriction. Termed the "brain sparing effect," this in-utero modulation of brain vascular resistance ensures cerebral perfusion is maintained [8]. Fetal ventriculomegaly, the most commonly identified abnormality of the fetal CNS, has been associated with elevated levels of mMPI [9].

Considering the relationship between fetal ventriculomegaly and elevated MPI levels, we hypothesized that other CNS pathologies might also have an impact on the myocardial performance index. Therefore, this study aimed to investigate whether there were changes in the myocardial performance index of fetuses with CNS pathologies without congenital heart diseases.

## Materials and methods

This cross-sectional study was conducted between April 2021 and December 2021 in the perinatology clinic of Van Yüzüncü Yıl University Hospital, Van, Turkey. All procedures followed were in accordance with ethical principles and the Declaration of Helsinki (as revised in Brazil in 2013). The local ethics committee approved the study protocol (Date/No: 04.2021/E-42884709-020). Written informed consent was obtained from all participating patients.

Study population

A total of 126 singleton pregnant women were included in this study. Sixty-three fetuses had fetal CNS abnormalities of open calvarium defects (acrania, anencephaly, and encephalocele) and closed calvarium defects (meningocele, hydrocephalus, and Dandy-Walker malformation). The remaining 63, matched for gestational age, constituted the control group, all of whom had a normal mid-trimester anatomical scan. Women with multiple pregnancies, those on medications other than multivitamins or iron supplements, and those with maternal conditions potentially affecting fetal cardiac functions, such as a history of QT syndrome, were excluded from the study. Fetuses were also excluded if they had multiple anomalies or any structural or functional cardiac abnormalities.

All data regarding obstetric history, gravida, parity, maternal age, gestational week, and history of previous children with cardiac abnormalities were recorded.

Ultrasound examination

All ultrasonographic assessments were conducted during the second trimester of gestation. Fetal biometric measurements were obtained, and in addition, images of the left ventricular outlet tract were captured. The MPI was calculated for all fetuses, ensuring the insonation angle ranged between 0 and 30 degrees. From the left ventricular cardiac waveform, peak velocities of the E, A, and S waves, along with the IVCT and IVRT, were measured. Subsequently, the mMPI was determined by using the following formula: (IVCT + IVRT) / ET [3]. Every fetal assessment was conducted thrice. A perinatologist with six years of experience performed ultrasonographic evaluations using a C2-9 convex transabdominal transducer on the Voluson E6 ultrasound machine (General Electric, Boston, MA). For each ultrasonographic measurement, intraobserver and interobserver variability had an intraclass correlation coefficient ranging from 0.83 to 0.95 [10].

Statistical analysis

Statistical analysis of the study was performed using IBM SPSS version 25 software for Windows (IBM Corp., Armonk, NY). Numerical data determined to be normally distributed based on the results of Kolmogorov-Smirnov tests are given as mean ± standard deviation (SD) values, while non-normally distributed variables are given as median (25th-75th quartiles) values. Accordingly, the Student's t-test and Mann-Whitney U test were used for comparisons between two groups, while ANOVA (post hoc: Bonferroni test) or Kruskal-Wallis H test (post hoc: Dunn test) were used for comparisons involving three or more groups. Categorical variables were presented as numbers and percentages, and comparisons between groups were performed using Chi-square and Fisher exact tests. Significance was accepted at a p-value <0.05 (*) for all statistical analyses.

## Results

The distribution of gravida, parity, abortion history, and gestational age was similar between the fetal CNS malformation and the control groups. Within the fetal CNS malformation group, anomalies predominantly included acrania (n = 27) and meningocele (n = 20). These were followed by hydrocephalus (n = 6), encephalocele (n = 4), anencephaly (n = 4), and Dandy-Walker syndrome (n = 2). The mean peak velocity of the E wave was similar between the fetal CNS malformation and the control groups. The mean peak velocity of the A wave was higher in the fetal CNS malformation group compared to the control groups (0.41±0.03 vs. 0.43±0.05 cm/s, P < 0.001), while the mean peak velocity of the S wave was lower (0.45±0.01 vs. 0.37±0.05 cm/s, P <0.001). The mean IVCT and mean IVRT were higher in the fetal CNS malformation group compared to the control groups, while the mean ET was lower. The mean mMPI was higher in the fetal CNS malformation group compared to the control groups (0.39±0.02 vs. 0.45±0.04 ms, P < 0.001) (Table 1).

**Table 1:** Demographic and clinical findings of the study population Data are shown as mean ±SD or median (25th–75th quartile). A: peak velocity of the A wave; E: peak velocity of the E wave; ET: ejection time; IVCT: isovolumetric contraction time; IVRT: isovolumetric relaxation time; mMPI: modified myocardial performance index; S: peak velocity of the S wave *P<0.05 indicates statistical significance.

Variables	Control group	Fetal CNS malformation group	P-value
	n = 63	n = 63	
Gravida, n	2 (1-3)	3 (1-4)	0.301
Parity, n	1 (0-2)	1 (0-2)	0.654
Alive, n	1 (0-2)	1 (0-2)	0.951
Abortus, n	0 (0-1)	0 (0-1)	0.758
Gestational age, weeks	20.30 ± 2.70	19.80 ± 2.40	0.274
Fetal cardiac parameters			
E, cm/s	0.25 ± 0.02	0.26 ± 0.04	0.563
A, cm/s	0.41 ± 0.03	0.43 ± 0.05	<0.001*
S, cm/s	0.45 ± 0.01	0.37 ± 0.05	<0.001*
ET, ms	172.80 ± 3.35	167.61 ± 4.00	<0.001*
IVCT, ms	28.50 ± 2.88	32.10 ± 3.20	<0.001*
IVRT, ms	38.84 ± 2.55	43.60 ± 3.90	<0.001*
mMPI	0.39 ± 0.02	0.45 ± 0.04	<0.001*

**Figure 1:**
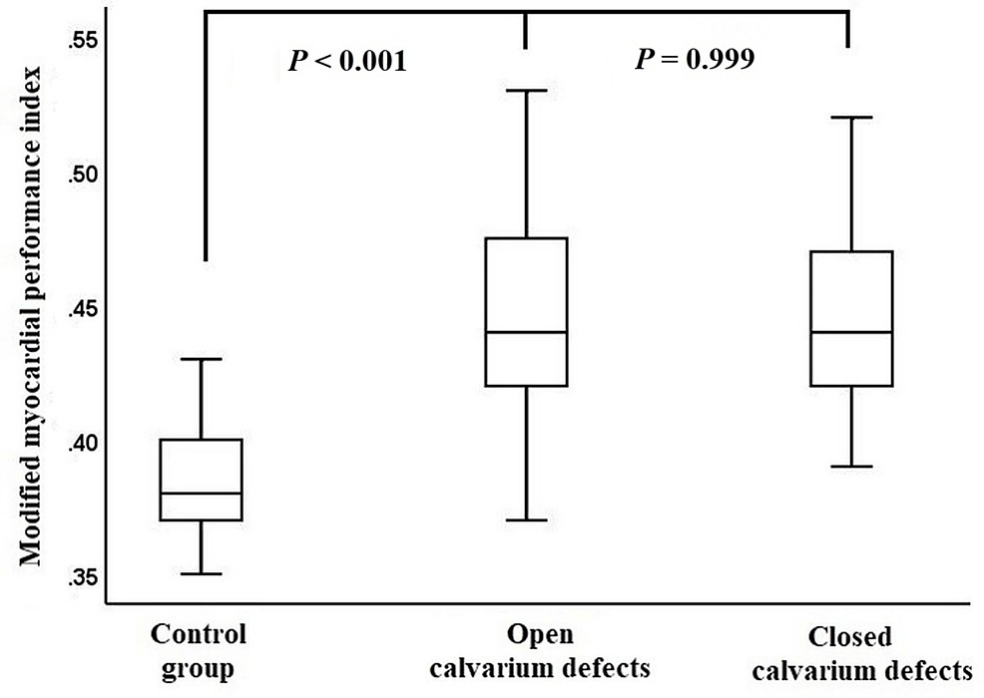
Distribution of the modified myocardial performance index in fetuses with closed and open calvarium defects compared to the control group

The mean peak velocities of the E and A waves in fetuses with closed calvarium defects were higher than in those with open calvarium defects and the control group. The mean peak velocity of the S wave was higher in fetuses with closed calvarium defects compared to those with open calvarium defects, but it was lower than in the control group. The mean IVCT, mean IVRT, and mean ET values were similar in fetuses with both closed and open calvarium defects. The mean mMPI value was similar for fetuses with both closed and open calvarium defects, but it was higher than in the control group (Figure 1, Table 2).

**Table 2:** Distribution of demographic and clinical findings in fetuses with open and closed calvarium defects compared to the control group Data are shown as mean ±SD or median (25th-75th quartile). The bold characters denote the group manifesting variances. A: peak velocity of the A wave; E: peak velocity of the E wave; ET: ejection time; IVCT: isovolumetric contraction time; IVRT: isovolumetric relaxation time; mMPI: modified myocardial performance index; S: peak velocity of the S wave *P<0.05 indicates statistical significance.

Variables	Control group	Calvarium defects group		P-value
		Open	Closed	
	n = 63	n = 35	n = 28	
Gravida, n	2 (1-3)	3 (1-4)	3 (1-4)	0.561
Parity, n	1 (0-2)	1 (0-2)	1 (0-2)	0.891
Alive, n	1 (0-2)	1 (0-2)	1 (0-2)	0.999
Abortus, n	0 (0-1)	0 (0-0)	0 (0-1)	0.915
Gestational age, weeks	20.30 ± 2.70	19.60 ± 2.10	20.10 ± 2.80	0.468
Fetal cardiac parameters				
E, cm/s	0.25 ± 0.02	0.24 ± 0.03	0.28 ± 0.03	<0.001*
A, cm/s	0.41 ± 0.03	0.42 ± 0.04	0.46 ± 0.05	<0.001*
S, cm/s	0.45 ± 0.01	0.35 ± 0.03	0.40 ± 0.07	<0.001*
ET, ms	172.80 ± 3.35	167.46 ± 4.30	167.80 ± 3.78	<0.001*
IVCT, ms	28.50 ± 2.88	32.31 ± 3.53	31.85 ± 2.87	<0.001*
IVRT, ms	38.84 ± 2.55	43.59 ± 4.11	43.62 ± 3.70	<0.001*
mMPI	0.39 ± 0.02	0.45 ± 0.04	0.45 ± 0.04	<0.001*

## Discussion

This is the first study to reveal differences in mMPI levels in fetuses with CNS pathology without congenital heart disease. The mMPI was higher in the fetal CNS malformation group than in the control group. However, it is similar in fetuses with both closed and open neural tube defects.

The MPI, a Doppler-based metric that measures both systolic and diastolic ventricular myocardial activity, is suggested to be a reliable indicator of global cardiac function [4]. While MPI is commonly employed in neonatal clinical evaluations, its application in fetal assessments is not as widespread [11]. Moreover, reference values can vary based on gestational age and ethnic origin. One of the large-sample studies reporting the normal ranges of fetal mMPI is based on cross-sectional data from 730 women conducted in Spain [12]. Another study was conducted in China, involving 2,081 women with singleton pregnancies [13]. There was a notable variance in reference values across the studies [12,13]. On the other hand, Hernandez-Andrade et al. reported that the reference value was 0.35 for the 19th week and 0.37 for the 39th week of gestation [5]. In the present study, the mMPI levels of the control group were consistent with the previously reported gestational age-specific reference ranges in Turkey [14]. Based on gestational age, elevated mMPI levels indicate diminished cardiac performance [15].

Numerous studies have shown that mMPI increases in pathological conditions such as intrauterine growth restriction, preeclampsia, gestational diabetes, twin-to-twin transfusion syndrome, and congenital heart malformations [16-20]. In a study on fetuses with isolated mild to moderate ventriculomegaly, it was reported that those with ventriculomegaly before 32 weeks exhibited a longer IVRT and higher mMPI compared to healthy control fetuses [9]. However, we did not come across any studies investigating mMPI in cases of fetal CNS malformations. In a dog model, surgically induced chronic hydrocephalus led to a notable decrease in cardiac output [21]. In a study conducted on fetuses that underwent myelomeningocele surgery, it was shown that stimulation of the spinal cord or nerve endings significantly increased mMPI during brain surgery [22]. These findings suggest that fetal CNS malformations may be associated with impaired cardiac function.

For CNS malformations with an open calvarium, the nerve tissues can be notably more vulnerable to damage and physical stimulation given their increased surface area in comparison to a closed calvarium [23]. Interestingly, there was no statistically significant variation in mMPI and its associated components when contrasting open and closed calvarium CNS malformation groups. On the other hand, within fetuses with closed calvarium defects, the peak velocities of the E, A, and S waves exhibited marked variations. These findings may indicate that different CNS malformations could have varying impacts on cardiac performance. Besides, among these CNS malformations, surviving neonates might be predisposed to developing heart failure. In neonates, the vein of Galen malformations arises from a pronounced AV shunt, resulting in high-output heart failure. Meanwhile, in infants, these malformations manifest with signs of macrocephaly or hydrocephalus [24]. At present, acrania and anencephaly are unviable anomalies; however, other CNS malformations such as meningomyelocele, Dandy-Walker malformation, and hydrocephalus are mostly viable and managed appropriately. Although the clinical importance and impact of higher fetal mMPI in the newborn period have not been elucidated yet, in such fetuses, the cardiac function of the newborn might need an evaluation to achieve better management.

This study has some significant limitations. Firstly, this study was performed in a single-center setting with a relatively small sample size. In the subgroup analysis, this might have caused the mMPI levels to show no significant difference between open and closed calvarium defects. Secondly, due to the recommendation of termination based on poor prognosis or unviability and the majority of the study group opting for termination, we could not conduct evaluations of pregnancy outcomes and newborn mMPI. Lastly, ethnic differences might influence the fetal mMPI. However, there is not a universal nomogram for mMPI because of the wide variations in various studies in the literature [16, 25]. Therefore, there is a need for large-scale prospective studies investigating the relationship between mMPI levels and CNS malformations, recruiting subgroups of different CNS malformations.

## Conclusions

This study showed that fetal CNS anomalies were associated with prenatal cardiac dysfunction. The fetal mMPI was higher in CNS malformations with respect to healthy fetuses. Although the clinical importance and impact of higher fetal mMPI in those fetuses have not been elucidated yet, those newborns might need a cardiac evaluation to achieve better management.
